# Photoplethysmography for demarcation of cutaneous squamous cell carcinoma

**DOI:** 10.1038/s41598-021-00645-4

**Published:** 2021-11-02

**Authors:** Simon Mylius Rasmussen, Thomas Nielsen, Sofie Hody, Henrik Hager, Lars Peter Schousboe

**Affiliations:** 1 Department of Otolaryngology at the Southdanish University Hospital, 7100 Vejle, Denmark; 2 Department of Electrical and Computer Engineering, Aarhus University, 8000 Aarhus N, Denmark; 3 Department of Plastic Surgery, 7100 Vejle, Denmark; 4 Department of Clinical Pathology, Vejle Hospital, 7100 Vejle, Denmark; 5 Department of Regional Health Research, University of Southern Denmark, 5000 Odense, Denmark

**Keywords:** Cancer, Surgical oncology, Biomedical engineering

## Abstract

A video processing algorithm designed to identify cancer suspicious skin areas is presented here. It is based on video recordings of squamous cell carcinoma in the skin. Squamous cell carcinoma is a common malignancy, normally treated by surgical removal. The surgeon should always balance sufficient tissue removal against unnecessary mutilation, and therefore methods for distinction of cancer boundaries are wanted. Squamous cell carcinoma has angiogenesis and increased blood supply. Remote photoplethysmography is an evolving technique for analysis of signal variations in video recordings in order to extract vital signs such as pulsation. We hypothesize that the remote photoplethysmography signal inside the area of a squamous cell carcinoma is significantly different from the surrounding healthy skin. Based on high speed video recordings of 13 patients with squamous cell carcinoma, we have examined temporal signal differences in cancer areas versus healthy skin areas. A significant difference in temporal signal changes between cancer areas and healthy areas was found. Our video processing algorithm showed promising results encouraging further investigation to clarify how detailed distinctions can be made.

## Introduction

### Squamous cell carcinoma and surgical challenges

Squamous cell carcinoma (SCC) is the predominant^1^ type of head and neck cancer, and the second most common form of cutaneous malignancy^2^. Surgical excision is the main treatment modality for SCC. Histology is performed to confirm the diagnosis, and complete removal is secured by particular examination of the resection margins^2^. If the cancer extends to the excision margins, there is risk of remaining cancer cells in the body, and such cancer positive margins are related to increased risk of locoregional recurrence^3^. The surgeon has to balance the need for complete removal of the skin cancer against the conservation of surrounding healthy tissue. The invasion of SCC into adjacent tissue can be a particular problem in the eyes, nose and ear regions^2^ where precise but still minimally-invasive surgery is needed.

The complete resection of SCC relies on palpation and visual inspection^4^. To ensure safety margins of the tissue removed, histological examination is performed^2^. Positive resection margins are at high risk for developing recurrent disease and are associated with poor prognosis in other head and neck cancers^4–7^.

Particularly in the head and neck region, the smallest possible excision margin is sought to avoid unneccesary mutilation of the skin and mucosa.

Increased blood supply through angiogenesis is a well known cancer characteristic^8,9^. Vascular endothelial growth factor is often expressed in malignant tumours and promotes angiogenesis and lymphangiogenesis, and it is associated with poor prognosis in different cancer types including head and neck SCC^10–12^. The resulting tumour vasculature is structurally and functionally abnormal^13^, and the perfusion highly heterogeneous^14^.

Noninvasive imaging techniques such as dynamic contrast-enhanced MRI evaluate tumour vasculature with the aim of predicting treatment response^15^ and monitoring treatment effects^16^. The obtained imaging parameters correlate with histological measures^15^.

Angiogenesis is also known and studied in SCC^17^, where it was found to have a different vascularity than other non-SCC lesions, such as basal cell carcinoma and melanoma^18–20^. An intra-operative imaging tool could help the surgeon visualize and guide the excision of the tumour with better margin control in real time^4^. At the moment, near-infrared fluorescent light is a technique employed to help ensure negative resection margins^4^. Drawbacks include the need for frequent probe repositioning and the high price of the system, which requires a reusable console^21^.

### Remote photoplethysmography

Remote photoplethysmography (rPPG) is a technique of contactless monitoring of human cardiac activities by detecting the pulse-induced subtle light signal variations on human skin surface using a multiwavelength RGB camera^22^. Photoplethysmography (PPG) first arose in the 1930’s, when Hertzman saw that the light reflected from the skin contained variations that could be captured with a photocell^23^. PPG has mainly been used in describing the signals from healthy individuals as well as developing algorithms for data extraction and analysis^23^. One recent study explored rPPG for localizing gastrointestinal SCC^24^. An rPPG system typically consists of a light source shining on the skin and an RGB camera sensor recording the reflected signal^22^. The green light channel is better for analysing blood flow as it contains the strongest PPG signal, based on the fact that haemoglobin absorbs green light better than red light and penetrates deeper into the skin than blue light^25^.

PPG has previously shown potential to be used in a camera-based intraoperative setting^26^, where data from the green channel could estimate the heart rate in 95.6% of cases. rPPG has been shown to be closely associated with cutaneous perfusion and sensitive to autonomic nervous activity^27^. A correlation of 0.91 has been shown between PPG and one of the main technologies for studying microcirculation, laser Doppler flowmetry, for endothelial activities^28^.

### Hypothesis

The hypothesis of this study was that the rPPG signal inside the area of a SCC is significantly different from the neighbouring healthy skin.

## Results

The results of the analysis are summarised in Table 1, see Methods section for details on calculating perfusion indexes. Perfusion indexes were calculated for different time periods of the same video recordings. The difference in perfusion indexes between biopsy and non-biopsy areas can be seen for different time periods.

**Table 1:** Average perfusion indexes (normalized 0–1).

Period	Confidence interval of difference	P
Full time frame	0.016-0.038	<0.001
10 seconds	0.001-0.038	0.044
5 seconds	−0.006-0.037	0.149
2 seconds	−0.005-0.028	0.161

Significant differences were observed for the full time frame and for periods of 10 seconds comparing mean flow values in biopsy vs. non-biopsy areas. So perfusion indexes are significantly different in the cancer tissue vs. the healthy tissue in the full time frame and in periods of 10 seconds.

## Discussion

### Strengths

Our results are similar to other recent results documenting differences between cancer and non-cancer areas based on rPPG changes^24^. This study targeted SCC and aimed to investigate differences in rPPG signal variation between healthy skin and cancer areas. We have demonstrated that significant differences in rPPG signal variations can be measured and documented with video recordings.

### Weaknesses

Light emitting diode (LED) surgery lighting was preferred, but in case of no LED, an alternative transportable LED lamp was used. This led to small noise levels in some recordings. The region of interest (ROI) selection was done freehand with reference to images showing the area by which the biopsy was taken and by excluding non-skin areas and frame edges.

The two dots used for image registration were set in far distance from the tumour but could in some cases be seen showing rPPG variation. This was dramatically decreased when low pass filtering the pixel signals, though very subtle movements caused persisting artifacts. The low pass filtration is in danger of removing relevant high frequency information, but this has not been demonstrated to be the case in our analysis.

### Future studies

Differences in signal flow between cancer and healthy tissue were observed not only in the total signal length, but also in shorter time segments of 10 seconds. This indicates that significant differences can be found in short video recordings.

In theory, a device to do video signal processing and visually present margins with a delay of 10 seconds might be usable and be of value in clinical practice. It could be implemented as an examination tool for use before or during surgery.

Another goal could be to do the flow analysis to visualize margin detection during resection. It would be beneficial to study the spatial resolution of the flow algorithm, which we will investigate in future studies.

## Methods

### Data and setup

Our study was conducted at the Department of Otolaryngology and the Department of Plastic Surgery (South Danish University Hospital). It was authorized by the University of Southern Denmark and approved by the Danish National Committee on Health Research Ethics and in accordance with the Helsinki Declaration. We chose to focus on SCC of the skin because it is easily seen, and also since finding a pattern in the rPPG signal changes might be more difficult if data represents multiple pathologies. Patients referred to hospital under suspicision of SCC and patients with biopsy determined SCC were enrolled in the study. Twentyone adults gave written informed consent to participate. Eight patients were excluded because histology disconfirmed SCC. Patient nr. 10 appears twice since two SCC tumours were recorded from this patient. Clinically relevant information about the volunteers was logged. We recorded the skin tumour in two recordings of 60 s. Table 2 summarizes characteristics of the selected patient group.

**Table 2:** Patient characteristics.

Nr	Age	Gender	SCC location
1	82	Male	Scalp
2	63	Female	Upper arm
3	53	Female	Lower leg
4	76	Male	Scalp
5	64	Male	Scalp
6	84	Male	Finger
7	89	Male	Back of hand
8	81	Male	Temple
9	83	Female	Foot
10	89	Female	Hand
10	89	Female	Neck
11	75	Male	Lip
12	85	Male	Ear
13	77	Female	Scalp

For video recording we used a mobile recording system consisting of an RGB camera (UI-3160CP-C-HQ Rev2.1 sensor, iDS, Germany) and a zoom lens (Navitar Zoom 7000, Navitar, USA). Recordings were done in 12 bit, 460 $\times$ 960 pixels. One recording of 60 s was done at 60 frames per second (fps) and one recording of 60 s was done around 300 fps depending on the noise and gain level, which were prioritized to be minimal.

**Figure 1:**
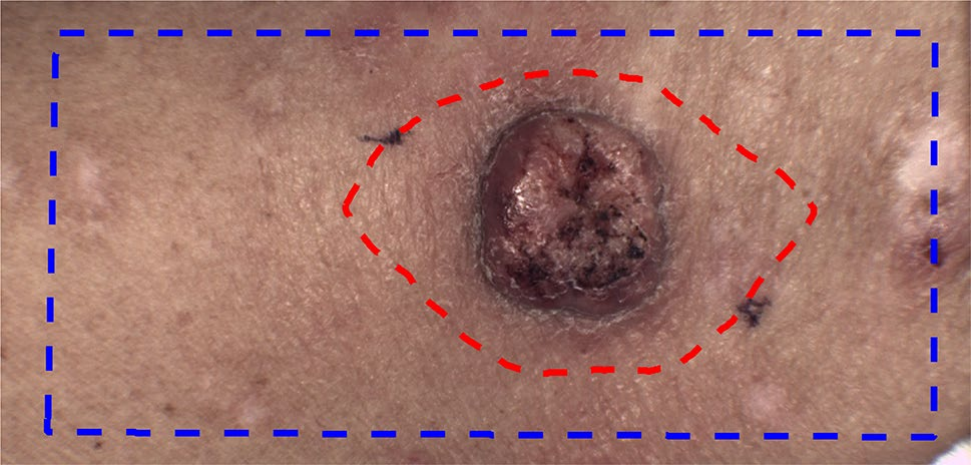
Example of masks. Blue line indicates the analysed skin area and the red line indicates the biopsy mask. The dots for image registration can be seen on both sides of the tumour.

To provide a basis for later image registration, two dots were placed opposed of the tumour, see Fig. 1. After focus and lightsources were adjusted, an X-rite ColorChecker was included in the recording for later image preprocessing (Bayer conversion and white balancing for eliminating possible emission differences). For each recording, the pulse, shutter speed, and noise/gain levels were noted. Afterwards either the surgeon’s marking of the biopsy to be removed or the actual skin minus the excision was recorded. These recordings were later used to mark the excision margin in the two 60 s recordings. Light sources were always LED, either by the surgical light or by a mobile LED lamp if no other LED light source were available.

### Case selection

After surgery the removed tissue was evaluated by a pathologist as to whether the tumour was SCC. Eight patients without SCC were excluded leaving 14 to be analysed.

### Video processing and registration

Data were recorded in a .seq format which was converted to uncompressed tiff-files. The tiff-files were white balanced and Bayer converted by using information from the frames containing the X-rite ColorChecker. The frames were combined to an uncompressed video format, .mj2. The video was then edited by removing the frames with the X-rite ColorChecker.

Based on the reasons given in the section on “Remote photoplethysmography”, processing and analysis were focused on data from the green colour channel. The remaining frames were registered by *rigid body* registration for the green colour channel using the Matlab rutines, imregconfig and imregister (MATLAB. (2020). version 9.8.0.1396136 (R2020a). Natick, Massachusetts: The MathWorks, Inc.).

### Flow analysis

**Figure 2:**
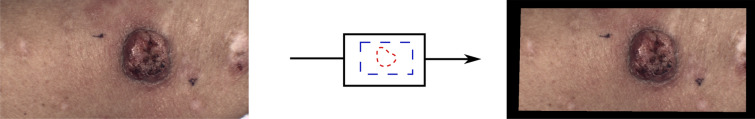
Removal of edges by area mask.

Videos recorded at 60 fps were selected. 60 fps was considered efficient since signals would later be low pass filtered at 20 Hz. For each video the area of the video was drawn as a mask, see Fig. 1 as example. Due to registration, the edges were cut away (see Fig.  2) and non-skin areas were also not selected. Secondly the resection margin was drawn in the video as a mask.

**Figure 3:**
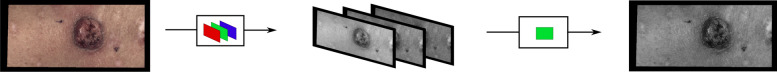
The green colour data were extracted from each frame of the video.

The green colour channel was then selected from the registered video, see Fig.  3.

**Figure 4:**
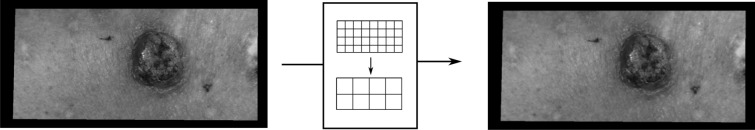
Each frame was divided in segments of $2 \times 2$ pixels in which the mean exposure replaced each of the four pixel values.

Each frame was divided in segments of $2 \times 2$ pixels in which the mean exposure replaced each of the four pixel values, see Fig.  4. This was done in order to reduce data amount.

**Figure 5:**
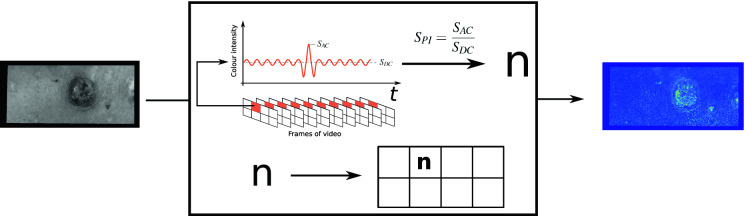
Illustration of the calculation of perfusion index for each single pixel in the video—in this example the period is the full time frame. Notice the marking of $S_{AC}$ and $S_{DC}$.

In order to remove noise and high frequency motions, the temporal variation of each pixel was low pass filtered with two methods. Initially by using a minimum-order filter with corner frequency of 20 Hz, a stopband attenuation of 60 dB and compensation of the delay introduced by the filter. 20 Hz was chosen to remove high frequency noise but still have plenty of frenquency band to frequencies of the cardiac cycle (1–2 Hz). Afterwards data were filtered with a moving average filter at size 5, which was chosen to effectively remove noise elements based on minimal movements during the recording. I.e., the variation in each pixel was analysed as independent signals. For a specific period (*T*) of time (*t*) and a given signal (*S*(*t*)), the ratio (perfusion index, $S_{PI}$) between mean ($S_{DC}$) and peak ($S_{AC}$) was found for *T*, see Eq. (1) and see Fig.  5.

1 $$\begin{aligned} S_{PI} = \frac{S_{AC}}{S_{DC}} \end{aligned}$$

**Figure 6:**
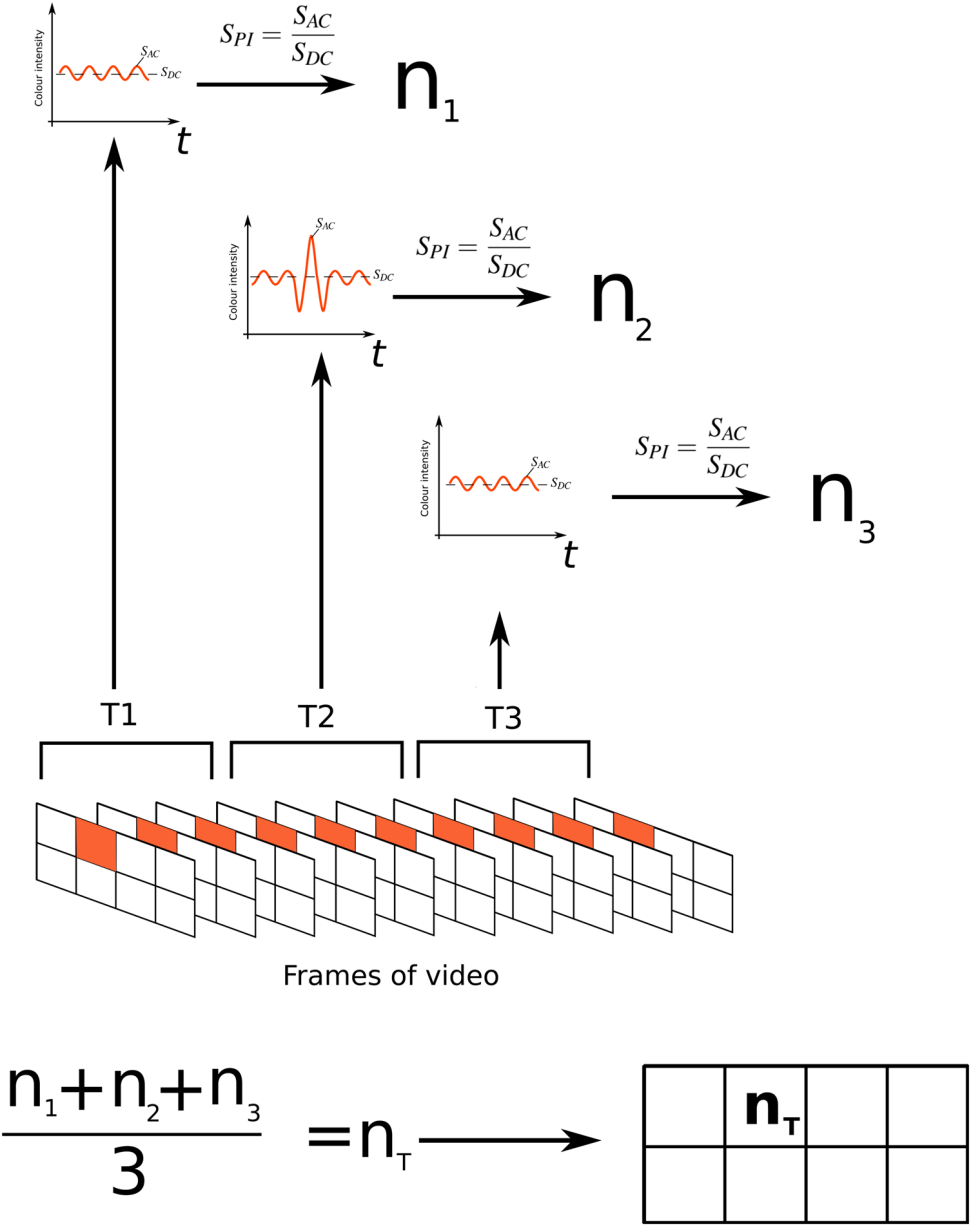
Illustration of the segmental analysis, in this example dividing the video into 3 segments. Each segment is analysed individually and a mean value, $n_T$, calculated in the end. Notice the individual markings of $S_{AC}$ and $S_{DC}$ for each segment.

Analysis of each signal was done in the full time frame, of segments of 10 s, of segments of 5 s and of segments of 2 s, see Fig.  6. When doing segmental analysis, the mean across the segments were calculated, see Fig.  6.

Figures 5 and 6 illustrate that the *n* value calculated over the full time frame and the $n_T$ value calculated as a mean of segments will not necessarily be the same. In the example the $n_T$ value will be smaller than *n*.

The image matrix consisting of a perfusion index value for each pixel signal was then normalized by converting the matrix to an intensity image containing values in the range 0–1. The normalized image was plotted in a new diagram according to the individual pixel positions. In the segmental analysis the normalization was performed for each period.

**Figure 7:**
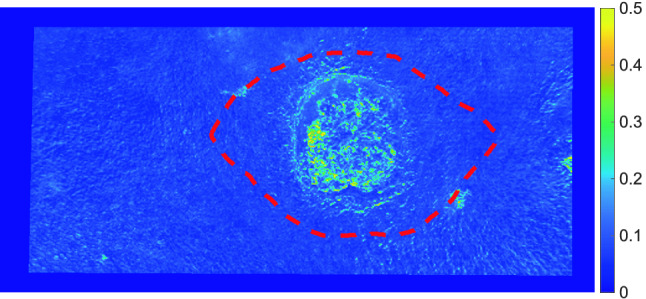
Example of flow chart. Resection mask is shown with red dashed line.

The result is a flow chart as depicted in Fig.  7.

The resulting flow charts were masked with the biopsy mask, and the average flow inside the mask was calculated. As an example see Fig. 7 for the areas of resection in the flow chart.

Likewise the average flow was calculated inside the mask defining the useful area, excluding the biopsy mask.

### Statistics

For each time period (full time frame, 10 s, 5 s and 2 s) a paired t-test was used to check for difference in means of perfusion index between indexes inside the biopsy masks and indexes outside the biopsy masks. P < 0.05 was considered statistically significant.

### Ethical approval

Our study was approved by the Danish National Committee on Health Research Ethics and in accordance with the Helsinki Declaration.
