# Author Correction: Photoplethysmography for demarcation of cutaneous squamous cell carcinoma

**DOI:** 10.1038/s41598-022-06856-7

**Published:** 2022-02-25

**Authors:** Simon Mylius Rasmussen, Thomas Nielsen, Sofie Hody, Henrik Hager, Lars Peter Schousboe

**Affiliations:** 1 Department of Otolaryngology at the Southdanish University Hospital, 7100 Vejle, Denmark; 2 Department of Electrical and Computer Engineering, Aarhus University, 8000 Aarhus N, Denmark; 3 Department of Plastic Surgery, 7100 Vejle, Denmark; 4 Department of Clinical Pathology, Vejle Hospital, 7100 Vejle, Denmark; 5 Department of Regional Health Research, University of Southern Denmark, 5000 Odense, Denmark

Correction to: *Scientific Reports* 10.1038/s41598-021-00645-4, published online 02 November 2021

The original version of this Article contained errors.

Table 1 contained several errors in the values given for “Full time frame”, “10 seconds”, “5 seconds” and “2 seconds” for “Confidence interval of difference” and “P”.

The incorrect and correct values appear below.

Incorrect:

**Table:** 

Period	Confidence interval of difference	P
Full time frame	0.019–0.070	0.0024
10 s	0.021–0.041	< 0.001
5 s	0.021–0.044	< 0.001
2 s	0.021–0.051	< 0.001

Correct:

**Table:** 

Period	Confidence interval of difference	P
Full time frame	0.016–0.038	< 0.001
10 seconds	0.001–0.038	0.044
5 seconds	− 0.006–0.037	0.149
2 seconds	− 0.005–0.028	0.161

As a result, in the Results section,

“Significant differences were observed regardless of time periods comparing mean flow values in biopsy vs. non-biopsy areas. So perfusion indexes are significantly different in the cancer tissue vs. the healthy tissue.”

now reads:

“Significant differences were observed for the full time frame and for periods of 10 seconds comparing mean flow values in biopsy vs. non-biopsy areas. So perfusion indexes are significantly different in the cancer tissue vs. the healthy tissue in the full time frame and in periods of 10 seconds.”

In addition, in the Discussion section, under the subheading ‘Future studies’,

“Differences in signal flow between cancer and healthy tissue were observed not only in the total signal length, but also in shorter time segments (10 s, 5 s, 2 s). This indicates that significant differences can be found in short video recordings.

In theory, a device to do video signal processing and visually present margins with a delay of 2 s might be usable and be of value in clinical practice. It may even be possible to differentiate in a time segment lower than 1 s leading to near real-time analysis. For this option, the time segments would have to be shortened to about 0.1 s^29^ to be of a real-time experience. If the pulsation is a necessary component for the differentiation, this will be a limiting factor of how short a time-frame can be achieved.

Another goal could be to do the flow analysis to visualize margin detection during resection. It would be beneficial to study the spatial resolution of the flow algorithm, which we will investigate in future studies.”

now reads:

“Differences in signal flow between cancer and healthy tissue were observed not only in the total signal length, but also in shorter time segments of 10 seconds. This indicates that significant differences can be found in short video recordings.

In theory, a device to do video signal processing and visually present margins with a delay of 10 seconds might be usable and be of value in clinical practice. It could be implemented as an examination tool for use before or during surgery.

Another goal could be to do the flow analysis to visualize margin detection during resection. It would be beneficial to study the spatial resolution of the flow algorithm, which we will investigate in future studies.”

The original Article has been corrected.

